# “I had your number on speed dial:” A qualitative study of patient experiences of dietetics care and managing nutrition during curative treatment of upper gastrointestinal cancer

**DOI:** 10.1371/journal.pone.0357580

**Published:** 2026-09-17

**Authors:** Irene Deftereos, Justin Yeung, Nicole Kiss, Vanessa Carter, Aurora Ottaway, Hollie Bevans, Danielle Hitch

**Affiliations:** 1 Department of Nutrition and Dietetics, Western Health, Melbourne, Victoria, Australia; 2 Department of Surgery, Western Precinct, Melbourne Medical School, The University of Melbourne, Melbourne, Victoria, Australia; 3 Western Health Chronic Disease Alliance, Western Health, Melbourne, Victoria, Australia; 4 Department of Colorectal Surgery, Western Health, Melbourne, Victoria, Australia; 5 Institute for Physical Activity and Nutrition, Deakin University, Geelong, Victoria, Australia; 6 Allied Health Research, Peter MacCallum Cancer Centre, Melbourne, Victoria, Australia; 7 Occupational Therapy, Western Health, Melbourne, Victoria, Australia; 8 Occupational Science & Therapy, Deakin, Geelong, Victoria, Australia; The Chinese University of Hong Kong, HONG KONG

## Abstract

**Background:**

Limited research exists on the implementation of nutrition care pathways to provide dietetics care in upper gastrointestinal cancer surgery, particularly from the patient perspective. This study aimed to understand patients’ experiences and perceptions of dietetics care provided via a nutrition care pathway, both before and after curative intent surgery. The secondary aim was to explore patients’ experiences of self-managing nutrition throughout their treatment trajectory and the impact on quality of life.

**Methods:**

This was a single-centre, prospective phenomenological study. Patients undergoing curative neoadjuvant therapy and surgery for oesophageal or gastric cancer were invited to participate in two semi-structured interviews: 1) in the period between completion of neoadjuvant therapy and surgery; 2) at 3 months post-surgical discharge. Questions pertaining to experiences and perspectives regarding dietetics intervention, nutritional status, symptoms, and quality of life were asked as per the interview schedule. Data were analysed and coded thematically using a phenomenological approach.

**Results:**

Thirteen participants were included (12 with oesophageal cancer and one with gastric cancer; mean age 63 years, 62% male). Four main themes were identified: 1) lived experiences of nutritional changes during treatment and recovery; 2) perceptions of nutrition and role of the dietitian; 3) feeding tubes and oral supplements as “the necessary evil”; and 4) impact of treatment and nutritional changes on daily life.

**Conclusions:**

Participants experienced significant nutritional challenges throughout the treatment trajectory, greatly impacting on daily life and psychosocial wellbeing. Participants found the dietetics care provided under the nutrition care pathway to be highly beneficial and acceptable, and dietitians were deemed an essential resource. The findings suggest that intensive, individualised nutrition care pathways, including oral and enteral supplementation, would be beneficial in similar clinical contexts, with peer support identified as an area for further resourcing and research.

## Introduction

Oesophageal and gastric cancers (collectively known as upper gastrointestinal (UGI) cancers) are associated with high morbidity and mortality, with the 5-year survival rates amongst the lowest of all cancer types [1]. Until recently, the curative treatment for oesophageal cancer has been resection preceded by chemoradiotherapy (the CROSS protocol) [2], followed by postoperative immunotherapy if residual tumour is noted on resection margins [3]. For gastric cancer, the curative treatment has been surgical resection with neoadjuvant and adjuvant chemotherapy [4]. These gruelling treatment regimens significantly impact on physical function, and patients often experience malnutrition, weight loss, muscle loss and gastrointestinal symptoms throughout preoperative treatment [5,6]. Post-surgical complications and changes in gastrointestinal anatomy often result in permanent symptoms affecting the ability to eat and drink normally, which can also exacerbate nutritional decline and negatively impact quality of life (QOL) [7].

Malnutrition and muscle wasting are independent risk factors for increased mortality, surgical complications, prolonged hospital stays, reduced chemo/radiotherapy tolerance and diminished QOL [8,9]. Therefore, European Society of Clinical Nutrition and Metabolism (ESPEN) guidelines recommend early preoperative nutrition support and long-term nutritional follow-up for UGI cancer patients undergoing curative intent treatment [8,10,11]. Guidelines also recommend that nutrition care should be delivered via structured nutrition care pathways to support optimal care delivery for these high-risk tumour groups [11]. However, nutrition care pathways are underutilised internationally, with a 2022 Australian study indicating fewer than 50% of hospitals have adopted structured nutrition care pathways for UGI cancer patients undergoing surgery [12].

At our health institution (Western Health, Melbourne, Australia), a nutrition care pathway aligned with evidence-based guidelines [8–10] has been established as standard practice for over five years. The nutrition care pathway outlines the frequency, type and timing of nutrition care based on clinical and nutrition risk assessments conducted throughout key treatment timepoints, with individualised nutrition care provided according to nutrition risk [13]. Dietetics care is also provided within the multidisciplinary surgical oncology setting. Our previous research has demonstrated positive effects of the nutrition care pathway in UGI cancer cohorts including improved access to care, lower rates of malnutrition at the time of surgery and better implementation of evidence-based recommendations for surgical nutrition care [12,13]. Furthermore, there was high acceptability among clinicians and patients [13,14]. However, qualitative data on patient-reported experiences and perceptions of dietetics care within the structured care pathway across the pre- and post-operative periods remains scarce. This gap limits the optimisation of health service delivery for this group of patients. Therefore, this study aimed to understand patients’ experiences and perceptions of dietetics care provided via a nutrition care pathway, both before and after curative intent surgery. The secondary aim was to explore patients’ experiences of self-managing nutrition throughout their treatment trajectory and the impact on QOL.

## Methods

### Study design

This was a single-centre, prospective phenomenological study. Ethics approval was sought and received on the 7 May 2019 (LNR/51107/PMCC-2019). Due to the implementation of COVID-19 restrictions mid-way through the study, most of the recruitment and interviews were conducted by telephone. This study is reported according to the Consolidated Criteria for Reporting Qualitative Research (COREQ) checklist (S1 File) [15].

### Nutrition care pathway

Details of the nutrition care pathway implemented at our institution have been reported previously [13,14]. The nutrition care pathway was developed using evidence-based guidelines and expert consensus from surgical oncology dietitians. The pathway provides risk-stratified dietetics interventions at key perioperative treatment stages, and outlines the recommended timing, type, and frequency of intervention. A key aspect of the pathway is that it is delivered within a multidisciplinary model of care, with implementation supported by the dietitian being integrated into the weekly multidisciplinary team meetings, and outpatient appointments co-located within the surgical oncology service. Patients received regular, individualised dietetics intervention from time of diagnosis until 3–6 months post-surgical discharge, according to evidence-based practice recommendations within the nutrition care pathway. The dietitians providing most of the dietetics care for patients in this study each had over 10–15 years of specialised experience in gastrointestinal surgical oncology.

### Participant eligibility and recruitment

Participants were eligible to participate in this study if they were ≥18 years, planned for curative intent neoadjuvant therapy and surgery for oesophageal or gastric cancer, and able to consent to participation by English language communication or with the presence of an interpreter. Study dietitians identified and approached eligible patients post-completion of neoadjuvant therapy to participate in the study, either face-to-face or via telephone. Participants were excluded if they were to receive palliative intent oncology therapy or surgery, or unable to consent. The intended, purposive sample size was fifteen participants. The sample size was based on feasibility considerations and consistency with prior qualitative studies conducted in similar clinical populations [7,16–18]. Recruitment commenced in July 2019, and the final interview was completed in September 2022. All participants provided written informed consent at recruitment, per the approved ethics protocol, and verbal consent was reconfirmed before each interview. Patients were also able to choose to have their primary carer participate in the interview if applicable, as research suggests that caregivers should be included as ‘co-clients’ in this population due to significant caregiver burden [19]. Consent included participation and publication of anonymised data from both patients and carers. All participants received the standard dietetics care according to the nutrition care pathway during the study.

### Demographic and clinical data

Demographic and clinical data were extracted from the medical records. This included age, sex, primary language spoken, social situation, tumour site/type, and treatment/surgical characteristics. Details of feeding tubes received preoperatively or postoperatively were also recorded. At the first interview, nutritional status was assessed verbally using the Patient-Generated Subjective Global Assessment Short Form (PG-SGA Short Form; S2.), a validated tool evaluating weight history, food intake, symptoms, and activities and function [20]. A score of 0–3 indicates no intervention required, a score of ≥4–8 indicates that the patient requires intervention by a dietitian and a score of ≥9 indicates a critical requirement for improved nutrition intervention and symptom control management. The PG-SGA Short Form is recommended for its suitability in telephone-based assessments [20]. Malnutrition at diagnosis was determined from clinical dietitian records using the International Classification of Diseases, 10th Revision Australian modification (ICD-10-AM) criteria, which defines malnutrition in adults as body mass index <18.5 kg/m^2^ or unintentional loss of weight of ≥5% with evidence of suboptimal intake resulting in loss of subcutaneous fat and/or muscle wasting [21]. Height and weight were obtained from the dietitian entries to calculate body mass index at the time of first interview. Quantitative demographic and clinical data were analysed descriptively using Microsoft Excel. Categorical variables were summarised using counts and percentages, while continuous variables were summarised using means and standard deviations (SD).

### Qualitative interviews

Semi-structured, qualitative interviews were conducted by the principal investigator. Participants took part in two interviews. The first interview occurred in the period between completion of neoadjuvant therapy and surgery; and the second interview occurred at approximately 3 months post-surgical discharge. The interviews followed a semi-structured guide with predefined questions tailored to each timepoint (pre-surgery and 3 months post-discharge, see S3 File), allowing flexible phrasing and probing to elicit detailed responses [22,23]. The guide included questions on dietetic care experiences, nutritional status, symptoms, and QOL. The first three participants’ interviews were initially conducted in person in a private clinic room, however COVID-19 restrictions required the remainder of interviews to be conducted over the phone. The interviews were approximately 45 minutes in duration. Caregivers were present for the entire duration of the interviews and the same guide was used with both patients and caregivers. There were no apparent differences between telephone and in-person modes of interviews in terms of duration of interview, or depth of participation.

### Data analysis

The interviews were recorded and transcribed verbatim for analysis by an external party. Transcripts were not returned to participants for review. Data were managed and analysed using Dedoose (Version 9.0.107, Los Angeles, CA: SocioCultural Research Consultants, LLC www.dedoose.com). Using a phenomenological approach, four transcripts (31%) were independently coded by two researchers (ID, DH); to develop an initial coding framework and support interpretive rigour and consensus. Participant and caregiver responses were analysed and coded together, with no differential weighting or separate coding applied. Once consensus regarding the coding framework and thematic structure was established, the remaining transcripts were coded by the primary researcher, with iterative discussions between researchers used to refine themes and sub-themes. Disagreements were resolved through discussion to achieve consensus, enhancing analytical rigour.

### Researcher positionality

The primary researcher (ID) holds a PhD and is a female dietitian and researcher who has previous experience and training in qualitative research. The primary researcher provided clinical dietetics outpatient care to patients in the study as part of the multidisciplinary surgical oncology clinic that services patients before and after their neoadjuvant therapy as per the nutrition care pathway. However, it is important to note that the team of surgical oncology dietitians providing care to this patient cohort consisted of at least four other specialist dietitians, none of whom participated in data collection or analysis. Patients received outpatient care from these other dietetics team members at certain periods and only received care from the neoadjuvant therapy dietitians during part of their treatment period. The researcher ensured that questions were asked about the dietetics care, rather than individual clinicians.

The research team also acknowledged that the interviewer’s clinical role may have influenced participants’ willingness to disclose negative experiences or criticisms. To mitigate this, reflexive discussions were undertaken during analysis to consider the potential influence of the researcher-participant relationship on data interpretation. Participants were aware that the researcher was completing the research with the goal of understanding more about patients’ experiences with the nutrition care pathway to improve care. The second researcher involved in coding (DH) is a female occupational therapist and researcher who holds a PhD and has expertise in qualitative research.

### Trustworthiness

To enhance trustworthiness, several strategies were used throughout data collection and analysis. A phenomenological approach prioritised participant-led narratives, with standardised probing questions used to support consistency across interviews. Independent coding of four transcripts by two researchers supported interpretive rigour and reflexive discussion during development of the coding framework. Iterative meetings between researchers were undertaken throughout analysis to refine codes, themes, and sub-themes, with disagreements resolved through discussion and consensus. Although transcripts were not returned to participants for review, rich participant quotations were included to support transparency and grounding of the findings in the data.

## Results

Thirty-two patients were eligible for the study during the study period, twenty-three were approached and fifteen participants were recruited to the study as per the intended sample size. However, two participants were withdrawn from the study; one could not participate in the second interview, and the other died prior to the second interview. The final number of participants who completed the study was thirteen. Caregivers were present for the interviews of eight participants (62%). As outlined in Table 1, most participants were male (62%), with a mean age of 63 years. All but one participant had oesophageal or gastro-oesophageal junctional tumours and had chemoradiotherapy as neoadjuvant treatment, followed by oesophagectomy surgery. For one non-English-speaking participant, a professional interpreter (language: Macedonian) facilitated the interview to ensure accurate communication. Malnutrition was present in 77% of the cohort at baseline according to dietitian assessment, and all these patients screened positively for requiring dietetics intervention according to PG-SGA SF score. The majority (92%) used a feeding tube either before and/or after surgery.

**Table 1:** Demographic and clinical characteristics of participants.

Characteristics	n = 13	
**Age (mean, SD)**	63	12
**Sex (n, %)**		
Male	8	62%
Female	5	38%
**Primary Language Spoken (n, %)**		
English	12	92%
Non-English	1	8%
**Tumour Location (n, %)**		
Oesophageal	8	61%
GOJ	4	31%
Gastric	1	8%
**Tumour Type (n, %)**		
Adenocarcinoma	13	100%
**Type of Neoadjuvant therapy received (n, %)**		
Chemotherapy and Radiotherapy	12	92%
Chemotherapy	1	8%
**Type of Adjuvant therapy received (n, %)**		
Immunotherapy	4	31%
No adjuvant therapy	9	69%
**Social Situation (n, %)**		
Lives with family/carer	11	85%
Lives alone	2	15%
**Surgery Received (n, %)**		
Ivor Lewis Oesophagectomy	10	77%
Minimally Invasive Oesophagectomy	2	15%
Total Gastrectomy	1	8%
**Anastomotic Leak (n, %)**		
Yes	5	39%
No	8	61%
**Body Mass Index (kg/m** ^**2**^ **) (n, %)**		
*<* 18.5	2	15%
18.5–24.9	5	39%
25.0–29.9	2	15%
≥ 30	4	31%
**Weight (mean, SD)**	72.4	16.3
**Malnourished as per dietitian at time of diagnosis (n, %)**		
Yes	10	77%
No	3	23%
**PG-SGA Short Form score at time of first interview (n, %)**		
0-3 (no intervention)	3	23%
4-8 (dietetics intervention required)	4	31%
≥9 (critical need for improved nutrition intervention/symptom management)	6	46%
**Feeding tube received and used pre-operatively (n, %)**		
Jejunostomy tube	3	23%
Nasojejunal tube	1	8%
Nasogastric tube	2	15%
No feeding tube received	7	54%
**Feeding tube received intraoperatively and used post-operatively (n, %)**		
Jejunostomy tube	12	92%
No feeding tube received	1	8%

### Thematic analysis

Four main themes were identified: 1) lived experiences of nutritional changes during treatment and recovery; 2) perceptions of nutrition and role of the dietitian; 3) feeding tubes and oral supplements as “the necessary evil”; and 4) impact of treatment and nutritional changes on daily life. As caregivers participated in 62% of interviews, the themes reflect both participants’ experiences and caregivers’ perspectives related to nutrition care and recovery. Themes, sub-themes, and codes are presented in Table 2, with illustrative quotes identified by participant number (P1–13), interview number (1–2), and whether the quote was from the participant’s caregiver.

**Table 2:** Results of thematic analysis.

Theme	Sub-theme	Codes
**Theme 1: Lived experiences of nutritional changes during treatment and recovery**	Symptom-driven barriers to eating	Fatigue affecting appetite and ability to eat
		Dysphagia and oesophageal pain
		Early satiety, abdominal pain, reflux
		Treatment side effects (nausea, vomiting, altered bowels, radiation oesophagitis, mucositis)
		Changing taste and food preferences
		Reduction in portion size, early satiety
		Eating slower after surgery
		Difficulty judging amount of food after surgery
		‘Nil by mouth’ after surgery
		Minimal symptoms during pre-surgical period
	Strategies to maintain nutrition	Texture modified diets (and dislike of these)
		Smaller, frequent meals/ Eating as a ‘full time job/chore’
		Tube feeding, oral supplements and food fortification
	Weight and muscle loss	Significant weight loss, muscle loss and deconditioning (before and after surgery)
		Weight as a measure of nutritional status
		Despite improving after surgery, not back to premorbid level
		Positive effect when weight going back up
	Emotional consequences of altered nutrition	Peer support would have been helpful
		Guilt and frustration about not eating enough, and about weight/muscle loss
		Hope that eating will improve with time
		Impact of weight loss on body image (negative and positive)
		Fear surrounding eating and complications after surgery
		Longing to eat normally
		Loss of confidence and enjoyment with eating
**Theme 2: Perceptions of nutrition and role of the dietitian**	Importance of nutrition for recovery	Awareness of importance of nutrition for recovery and surgical outcomes, weight, muscle, and strength maintenance
		Shift in mindset regarding ‘healthy eating’ versus adequate calories and protein
		Focus on maintaining/improving nutrition to get back to doing usual tasks
	Dietitians’ multifaceted support	Dietitians as an essential part of the medical treatment
		Dietitian was approachable and easily contactable
		Advice provided helped with nutritional status, recovery, and symptom management
		Individualised, practical advice is important and valued
		Emotional support and confidence from dietitian’s regular review
		Seeking reassurance that their experience is ‘normal’/ Help navigating the “unknown”
	Dietitians as facilitators of Care	Surgeons valued the dietitians as a core member of the surgical team
		Dietitian as the ‘only point of contact’
		Dietitians facilitating medical reviews for complications or questions
**Theme 3: Feeding tubes and oral supplements “the necessary evil”**	Challenges of feeding tubes and oral supplements	Pain associated with feeding tube
		Tube-related complications- leakage, blockage, migration
		Sleeping issues related to overnight feeding
		Impact of feeding tube on daily activities
		Taste fatigue and dislike of oral supplements
	Nutritional benefits	Stability during treatment due to using oral supplements/feeding tubes
		Awareness of importance of taking oral supplements
		Recognition of the benefits of tube feeding for maintenance of nutritional status
		Recommending tube feeding to others
		Enjoyment of oral supplements
	Emotional aspects	Emotional relief when using tube feeding
		Looking forward to tube removal
		Worry about maintaining enough oral nutrition after tube removal
		Frustration regarding reliance on oral supplements instead of food
**Theme 4: Impact of treatment and nutritional changes on daily life**	Limited function and participation	Impact of weight and muscle loss on ability to perform daily tasks and meaningful occupations
		Impact of treatment and surgical complications on ability to perform daily tasks and meaningful occupations
		Extreme fatigue
	Reliance on social support	Heavy reliance on support from family and friends
		Difficulty accepting help and seeing others perform their usual occupations
	Adjustment to new routines	New routine of eating habits post-surgery
		Adjustment of work routines for new required eating habits
		Positive effects of weight loss on other comorbid conditions
	Social and emotional impacts	Reduction in social activities and invitations/ feeling ‘left out’ at mealtimes
		Stress regarding eating in social situations
		Positives when able to participate in meals with others
		Adjusting expectations of recovery after surgery
		Family/carer stress regarding cooking and eating
		Resilience and engagement in own treatment and recovery
		Recognition and acceptance of the ‘price paid’ for cure

### Lived experiences of nutritional changes during treatment and recovery

Participants reported significant nutritional challenges and symptoms throughout their treatment trajectory, driven by tumour-related symptoms, neoadjuvant therapy side effects, and post-surgical anatomical changes or complications. Four sub-themes emerged: Symptom-driven barriers to eating, strategies to maintain nutrition, weight and muscle loss, and emotional impact of altered nutrition.

#### Symptom driven barriers to eating.

During neoadjuvant therapy, participants reported oesophageal obstruction, dysphagia, poor appetite, and fatigue as primary barriers to oral intake. One participant described, “*Everything I ate seemed to just sort of get stuck halfway and then come straight back up again. And in the end I just pushed the plate away,”* (P8, Interview 1). Symptoms including vomiting and regurgitation often necessitated liquid diets or feeding tubes: *“I couldn’t actually eat food unless it was all liquified … and then you just got sick to death of eating the same thing”* (P1, Interview 1). Radiation oesophagitis and nausea further limited intake: “*so big heartburn where I just couldn’t eat at all. I just, I went right off my food again. I couldn’t get anything down”* (P12, Interview 1). Carers echoed this, observing the impact of exhaustion on their loved ones: *“He was too tired to eat”* (P11, Interview 1, carer). Three participants reported minimal preoperative symptoms despite significant weight loss at diagnosis. Post-surgery, participants faced ongoing postprandial fullness, regurgitation, reflux, and dumping syndrome: *“If I eat too much, I feel nauseous and have diarrhoea”* (P8, Interview 2).

#### Strategies to maintain nutrition.

To combat nutritional decline, participants used texture-modified diets, frequent snacking, and food fortification, though these were often disliked and challenging to uphold: *“I couldn’t stomach it the pureed diet, the taste of it, the texture, just everything about it was a chore.”* (P8, Interview 2). Post-surgery, many participants reported fear about progressing to normal textures due to concerns about complications: “*after two weeks eating puree only… I was scared to start eating normal foods”* (P5, Interview 2). Most reported significant loss of appetite post-surgery: “*when I come out of surgery it was, you were never hungry, you never felt hungry.”* (P4, Interview 2). Participants described a ‘trial and error’ process of adjusting to altered gastrointestinal anatomy: “*So instead of, like at the start I was pushing to see what I could eat and now I’ve realised what I can and what I can’t eat. I’ve been steering clear of all those”* (P6, Interview 2). The need for smaller, frequent meals to prevent symptoms was challenging for some: “*she wanted to only eat 3 meals a day. And – and of course she can’t, she has to have 6 or 8 meals”* (P1, Interview 2, carer). Carers played a critical supportive role, using strategies such as scheduling meals to ensure intake: *“I set an alarm... to get the three meals into him”* (P11, Interview 2, carer). By the second interview, most participants tolerated normal textures but still avoided trigger foods, and many described eating as a necessity rather than a pleasure: *“I’m eating because I need to, not because I enjoy it”* (P10, Interview 2).

#### Weight and muscle loss.

Most participants experienced significant weight and muscle loss before diagnosis and/or during treatment: *“I went from 74 to 50 [kg]”* (P12, Interview 1). Despite this, weight was often stable or improving within several weeks of neoadjuvant therapy completion: *“Yeah well my weight went down – a fair bit. But I put a few kg back on again too”* (P11, Interview 1). Three patients reported minimal nutritional issues during neoadjuvant treatment, although these patients all had presented with significant weight loss at diagnosis. Loss of weight and strength remained prominent in the second interview, particularly for the patients who had post-surgical complications: *“I would look in the mirror and I was like someone coming back from the First World War”* (P2, Interview 2). All participants reported significant post-surgery loss of strength, particularly immediately after discharge: *“strength I think is down, I get tired quite quickly”* (P7, Interview 2). By the second interview, their weight was stabilising or improving but was yet to return to premorbid levels: *“I’m doing things, but some weakness goes over my body”* (P5, Interview 2).

#### Emotional impacts of symptoms and altered nutritional status.

Symptoms during both neoadjuvant treatment and post-surgery caused significant emotional distress: *“You want to eat but you can’t, it’s a bit depressing. And generally just felt, you know, really sort of down”* (P10, Interview 1). Several participants reported being scared to eat because the symptoms were so significant: *“I was scared to eat because of the burning and the difficulty, and that was probably the hardest thing to get over”* (P6, Interview 1). Changed body image due to severe weight loss was distressing for several participants, with many having to adjust their expectations regarding weight and muscle gain after surgery: *“yeah, I felt pretty bad. And thought, I’ll never put this weight on”* (P8, Interview 2). Carers also experienced distress from their observations of physical decline: *“His ribs were sticking out... he looked awful”* (P11, Interview 2, carer). By the second interview, many patients had resigned themselves to altered eating habits and gastrointestinal changes. Furthermore, patients and carers often desired peer support to normalise their experiences: *“We really need a group... to talk about things”* (P1, Interview 2).

### Perceptions of nutrition and dietetics role

Participants viewed nutrition as a critical component of their treatment and recovery, and valued dietitians as essential members of the multidisciplinary team. Three sub-themes emerged: importance of nutrition for recovery, dietitians’ multifaceted support, and dietitians as facilitators of care.

#### Importance of nutrition for recovery.

Participants recognised adequate caloric and protein intake as vital for recovery and strength: *“If I’m stronger, I’ll go through [surgery] easier”* (P5, Interview 1). This awareness persisted post-surgery, with nutrition viewed as central to QOL: “*You know they can operate on you, and they can do anything. But if you’re not eating, and the right foods, or you’re not eating at all, where would you be? You’d be back to square one…. nutrition the most key factor before and the operation, because it’s just helping you get back to life”* (P10, Interview 1). Some participants adjusted their prior beliefs about “healthy eating” by prioritising high-calorie diets, but this shift in perception was difficult: *“I had to reverse my mind... to gain weight”* (P9, Interview 1). Carers also emphasised the role of nutrition in recovery: “*I think it’s very important, because he needs the strength and the nutrition especially to go through such a big surgery.. to pull through quicker”* (P3, Interview 1, carer). Even participants with minimal preoperative symptoms aimed to optimise nutrition before surgery.

#### Dietitians’ multifaceted support.

Dietitians were perceived as indispensable, providing practical and individualised advice: *“Most things suggested have been achievable”* (P2, Interview 2). Surgeons also stressed the importance of the dietitian to patients: “*I remember the surgeon saying that, you know the first thing he said was, “I want you to see the dietitian because we work really closely with them in these cases”, and yeah you could tell that it was important”* (P8, Interview 1). Participants valued the dietitian’s emotional support and reassurance: “*So, you know she sat him down and she talked to him and asked what his symptoms were, and you know she was very empathetic”* (P3, Interview 1, carer). Patients and carers described the dietitian as a reassuring constant throughout treatment: “*I like to know that somebody’s got my back, and the dietitians have”* (P1, Interview 2). Reassurance from dietitians regarding their progress and what was ‘normal’ or ‘expected’ was welcomed by all participants and carers: *“We would hang out for those moments where the dietitian team would reassure us that it was okay, it was normal”* (P9, Interview 1). Regular interactions with dietitians boosted confidence, including telephone consultations undertaken during COVID-19 restrictions: *“They reassure me I’m on the right path”* (P8, Interview 2). Carers also appreciated dietitians’ accessibility and efforts to support both them and their loved one: *“No matter what questions I had, she’d find out”* (P3, Interview 2, carer). Dietetics support was perceived as particularly critical post-discharge, when many participants felt vulnerable navigating nutritional challenges: *“I have your number in my speed dial so to speak, any time I had a problem I knew I could ring you and ask you, so yeah I always felt like I was in touch”* (P8, Interview 2).

#### Dietitians as facilitators of care.

Dietitians were also perceived as conduits for access to the surgical team, with an important role in coordinating care and communication: *“The dietitians have been a sounding board... a go-between sometimes”* (P2, Interview 2). Surgeons were also reported to reinforce this role: *“I said, what if something goes wrong? And the surgeon said, ‘Ring the dietitians, they’ll let us know’”* (P2, Interview 2). This integration within the multidisciplinary team enhanced care delivery, though two participants noted they found it easier to communicate with the dietitians than with medical staff. Carers also really valued this coordination: *“I always felt in touch”* (P8, Interview 2, carer), highlighting dietitians’ role in reducing health service access barriers.

### Feeding tubes and oral supplements “the necessary evil”

Participants and carers described feeding tubes and oral supplements as essential yet challenging for maintaining nutrition. Three sub-themes were identified: challenges of feeding tubes and supplements, their nutritional benefits, and emotional impacts.

#### Challenges of feeding tubes and supplements.

Many participants experienced complications with feeding tubes, including leakage, blockage, and discomfort: *“The pad…is pretty soaked…as it’s leaking and in the morning it’s very yellow…and I change the dressing”* (P2, Interview 2). Nasal tubes caused irritation, which prompted early removal for one participant: *“It was constantly irritating my throat... I couldn’t handle it”* (P10, Interview 1). Overnight feeding also commonly disrupted sleep: *“I was worried about pulling it out while asleep”* (P7, Interview 2). Carers found supporting their loved one with tube management very stressful: *“He was scared... ‘Go to emergency!”* (P5, Interview 2, carer). Those patients who had a feeding tube prior to surgery reported finding it easier to manage home feeding after surgery and that this was a significant benefit to them: *“..but with me it was already there, used to it, was already sleeping with it, but I’d hate to go straight after surgery and have it”* (P4, Interview 2). *Use of* oral supplements often resulted in taste fatigue and fullness: *“You get sick of the same thing day in, day out”* (P1, Interview 1), which in turn discouraged solid food intake. However, two participants reported minimal issues with supplements, and found them manageable: “*They weren’t hard to manage, I just drank them”* (P5, Interview 2).

#### Nutritional benefits.

Despite challenges, participants understood that feeding tubes are critical for nutritional support: *“It stabilised me because I was dropping quite quickly”* (P1, Interview 1). Tubes and supplements were also seen as vital when oral intake was insufficient, particularly post-surgery: “*A necessary evil I’d say. Yeah, it was uncomfortable…but I think I needed it because otherwise I don’t think I would have gained weight at that time”* (P8, Interview 2). Carers also appreciated the benefits of nutritional supports: *“The feed is keeping the weight on him”* (P3, Interview 2, carer). For the majority, the benefits outweighed the challenges, as described by one participant: *“Without the tube feeds, I would have been too weak… so my recommendation is for anybody do it”* (P6, Interview 2). Participants also recognised the importance of taking oral supplements when they could not maintain adequate food intake due to the various symptoms or complications described: “…*but I realise I’ve got to do it at the moment... I’d probably be pretty weak because I probably couldn’t survive on the food I’m having at the moment…. I’ve just got to do what I can to keep up with the supplement drinks and also try and eat more food”* (P2, Interview 1).

#### Emotional impacts.

On the positive side, feeding tubes helped to alleviate the anxiety participants felt about inadequate oral intake: *“It took the pressure off... I didn’t have to stress”* (P9, Interview 2). However, the thought of tube removal provoked mixed feelings: *“I thought, it’s a crutch, I’ve got to get away from it”* (P8, Interview 2). Carers were also anxious about the prospect of weight loss following tube removal: *“I’m worried the weight’s going to go down again”* (P3, Interview 2, carer). Dietitian support was crucial for managing these concerns and expectations as part of their role as care facilitators: “*we probably almost would have wanted to give up hey if we never got that support. It’s true. We had some hard days obviously”* (P12, Interview 2, carer).

### Impact of nutritional changes and treatment on daily life

Treatment and nutritional challenges profoundly affected the daily life of participants, having physical, social, and emotional consequences. Four sub-themes emerged: limited function and participation, reliance on social support, adjustment to new routines, and social and emotional impacts.

#### Limited function and participation.

Participants experienced a reduced ability to perform daily tasks due to weight loss, muscle weakness, and treatment side effects: “*I do restrict myself a bit, if I feel tired, I sit down. I don’t carry on”* (P7, Interview 2). This caused significant frustration and disappointment: *“You can’t do what you normally do”* (P10, Interview 1). Complications, such as infections or prolonged ‘nil by mouth’ periods, also compounded their functional limitations: *“I just laid around in bed”* (P12, Interview 1). Carers also identified this problem, which impacted their own lives in diverse ways: *“He’s not doing any of that [usual tasks], it’s a different life”* (P2, Interview 2, carer). By the second interview, strength was improving for all participants but had not returned to pre-surgical levels: *“I’m doing more... but the journey’s not over*” (P12, Interview 2, carer).

#### Reliance on social support.

Participants depended heavily on their family and friends for both practical and emotional support: *“Everyone... gave us a hand”* (P11, Interview 2). Their support was perceived as critical to their ongoing health and wellbeing: *“Without a carer to monitor what’s going on, I’d hate to think”* (P1, Interview 1). However, accepting help was difficult, particularly for previously active men: “*I’ve never been the sort of person to ask for help unless I really, really, really need it”* (P10, Interview 1). Some carers also experienced burden or negative impacts from providing assistance: “*I’d get a little bit frustrated when he wouldn’t eat you know I just kept thinking to myself, well you know you really got to eat you know you’ve got an operation coming up and you know you’ve got to keep your nutrition”* (P3, Interview 2, carer). This sometimes impacted the carer/patient relationship: “*My wife has to chase me around a bit to make me eat”* (P7, Interview 2), particularly with regard to cooking family meals *“she just cooks something for herself at night – most of the time now she’ll say “What do you want?” And I’ll say “Look I don’t feel like eating…”* (P2, Interview 2).

#### Adjustment to new routines.

Post-surgery, participants needed to adapt to new eating habits, requiring smaller, frequent meals: *“I used to have decent-sized meals, now it’s small meals”* (P10, Interview 2). These changes impacted on longstanding habits and routines, including employment for some: *“I can’t eat while driving [trucks] anymore”* (P13, Interview 2). However, some participants perceived positive outcomes from these changes, such as reduced reliance on heart medication due to weight loss. Carers were important facilitators of these adjustments: *“I set his breakfast at lunchtime to fit his schedule”* (P11, Interview 2, carer). The need for ongoing adaptations underscored the value of ongoing dietary guidance within health services in the post-surgical phase.

#### Social and emotional impacts.

Physical limitations were frustrating and upsetting for patients “*I know it sounds stupid, but you find it frustrating that you can’t do what you would normally do”* (P10, Interview 1). Nutritional limitations resulted in greater social isolation for some participants: *“I’ve never realised how much s ocialising is revolved around food. A lot of people go for dinner and stuff like that, they don’t include us anymore”* (P8, Interview 2). Eating in public provoked anxiety and feelings of vulnerability in case there was a problem: *“You’re worried something’s going to get stuck”* (P13, Interview 2). However, positive experiences of social meals felt very rewarding: *“It was nice to have the same meal as my son”* (P10, Interview 2). After surgery, patients remained engaged in their own treatment and recovery, reflecting a resilient but ‘resigned’ attitude “*yeah it is what it is, and that’s it”* (P11, Interview 2), accepting that the ongoing challenges faced were the price paid for ‘cure’ of their disease “*well you have to learn to live with it and just accept it and move on”* (P12, Interview 1).

## Discussion

In this qualitative study, patients reported a high number of nutrition-related symptoms and complications during neoadjuvant therapy and post-surgery, consistent with previous evidence showing 40–60% malnutrition rates in patients with UGI cancer [5,6,24]. Despite these challenges, participants and carers showed strong awareness of nutrition’s benefits, adopting strategies to optimise their status and reduce symptoms. However, this often altered their relationship with food, diminishing enjoyment and causing social isolation in some cases.

Similar themes have emerged in previous qualitative studies [16,17,25–27]. A qualitative study of 10 oesophageal cancer patients undergoing neoadjuvant treatment with surgery, or definitive chemo/radiotherapy, described their significant difficulties maintaining nutrition during treatment and after surgery [28]. Malstrom et al. (2013) interviewed 17 patients 2–5 years post-oesophagectomy or gastrectomy, with participants reporting ‘eating as an obligation’ in the context of significant ongoing gastrointestinal symptoms and weight loss [27]. Similarly, a recent study by Vaccaro et al. (2025) of 10 post-gastrectomy patients described disruption to body image, adaptation to new eating patterns whilst mourning past pleasures, and social isolation at mealtimes [26]. However, some participants in previous studies felt positively about their weight loss [17,26,28]. While weight remains a key marker of nutritional status, participants in this study also identified muscle and strength loss, supporting calls for greater emphasis on muscle as a prognostic indicator in cancer, particularly for patients with higher body mass index [18,29]. These findings therefore suggest incorporating muscle assessments into nutrition care pathways to enhance personalised care.

Best practice guidelines recommend supplemental tube feeding during chemo/radiotherapy and post-surgically for patients unable to meet oral requirements, with some evidence for prophylactic feeding tube insertion during chemoradiotherapy [30]. The challenges associated with feeding tubes faced by participants in this study -- like leakage, blockage and sleep disruption -- also align with findings from previous qualitative studies with UGI cancer, and are therefore not isolated [31–33]. However, like this study, other research with UGI, head and neck, and pancreatic cancer patients confirm they often view the nutritional and emotional benefits as outweighing the drawbacks [31–35]. This perspective counters clinician assumptions about tubes being ‘invasive’ or ‘last resort,’ indicating a potential role for them earlier in nutrition pathways. Our participants also had challenges with oral supplements, which were consistent with findings from other UGI cohorts [16,28,32,36]. However, unlike prior research, there was high compliance with this intervention reported from our participants. This may reflect the impact of individualised dietitian troubleshooting and support via the nutrition care pathway, suggesting a potentially cost-effective strategy for health services to improve adherence and reduce complications [37].

A high level of carer support was deemed essential by participants in this study, particularly in the post-surgical period. A recent systematic review [19] also highlighted significant burden for the carers of people with UGI cancers, particularly around increased responsibility for managing nutritional complications and subsequent dietary alterations. Support from other family members and friends was also valued in this study, however it was difficult to accept in some cases. Several participants commented on the need for peer support throughout their journey to “normalise” their experience. Calls for greater peer support for patients also reflect the findings of previous studies [16,17,31], and this form of support may also be helpful for reducing isolation for carers [19]. Peer support interventions, ideally embedded into the nutrition care pathway, are therefore recommended for this population, with a focus on nutrition and QOL.

The nutrition-related difficulties experienced by people with UGI cancers necessitate specialised and regular dietetics care, enabled by process-driven, evidence-based nutrition care pathways [13,17,31]. Individualised symptom management advice from a ‘confident and supportive’ dietitian is also identified in other studies as an important support for patients [31,38]. Both patients and carers in this study confirmed dietitians were an invaluable resource throughout their treatment trajectory who facilitate achieving nutritional optimisation. However, previous research indicates access to dietitians is not enough, as patients may still experience unmet needs or timely, personalised care and practical information, particularly in the initial postoperative period [16,28,31]. Participants in this study found their dietetics care highly acceptable and personalised, further supporting the delivery of this care as part of systematic and comprehensive nutrition care pathways. Consistent with previous research, dietitian reviews completed via telehealth due to COVID-19 restrictions were also acceptable to the participants, providing greater flexibility in the delivery of these services [14,38], and because of this we have continued to offer telehealth appointments within our current clinical nutrition care pathway.

Participants reported many additional psychosocial benefits to regular dietetic input throughout their treatment, including support for both emotional health and self-confidence, as described in another Australian study investigating intensive dietetics care [38]. Our participants felt the dietitians helped them understand what was ‘normal’ or not, which was identified as an unmet need in Sadeghi et al. (2025) [16]. A key finding from this study was the role of dietitians as ‘conduits’ to the surgical team, which was also reported by the dietitians themselves [14]. Two previous qualitative studies reported that patients were frustrated at the lack of medical professional contact and support post-surgery, leading to unanswered questions and a lack of information about symptom control unless clinical nurse consultants filled this gap [17,31].

The study site did not have clinical nurse consultant positions for UGI cancer patients at the time of the study, and therefore the dietitian assumed additional supportive and coordination responsibilities within the multidisciplinary team. As a result, some findings regarding the role of the dietitian may not be fully transferable to institutions with established clinical nurse consultant roles. Close working relationships and mutual professional respect between surgeons and dietitians appeared to support this extended role, in contrast to experiences reported in previous Australian and international studies [16,17,31]. The findings also highlight the importance of effective dietitian integration within the multidisciplinary team for successful implementation of the pathway. Key features that may support transferability to other settings include dietitian participation in weekly multidisciplinary team meetings, which facilitates timely identification and referral of high-risk patients, and co-location of dietetics appointments within the surgical clinic setting to support coordinated and timely care valued by patients and caregivers.

In this study, the emotional challenges of both treatment and adjustment to a “new normal” post-surgery featured heavily in patient and carer interviews. Many participants felt frustration, anxiety, depression and sadness about the loss of their previous abilities to eat, drink and enjoy food. The emotional aspects of coping with long-term changes after surgery also feature in several other studies [17,26,28], which also note a feeling of ‘resignation’ towards their new life post oesophageal cancer surgery. Improved models of care including psychological interventions to address these emotional impacts would also be beneficial for longer term care.

### Strengths and limitations

This is the first study to qualitatively explore patients’ perspectives of their nutrition, and dietetics care received via a nutrition care pathway, across the extended curative treatment trajectory. As interviews were conducted longitudinally before and after surgery, evolving experiences and perspectives were captured over time. Two researchers with qualitative research experience completed data coding and data analysis using a rigorous process. In this study, carers were engaged in over half the interviews, which provided valuable additional context to the patients’ perspective. However, due to recruitment challenges contributed to by the pandemic, the sample lacked diversity with regard to tumour location and ethnicity. Almost all participants had oesophageal or GOJ tumours, nearly all received chemoradiotherapy and oesophagectomy, and only one non-English-speaking participant was included with an interpreter. This requires consideration when interpreting the breadth of the themes**.** Two patients also completed their final interviews 2 and 3 months later than the remainder of the group due to being unwell, whilst two patients withdrew from the study resulting in a lower sample size than initially intended. The single-centre design may restrict generalisability, as findings could have been influenced by institution-specific factors, including the established care pathway and multidisciplinary team dynamics. Findings relating to the role and value of dietitians may have been shaped by the institution’s established pathway and close multidisciplinary relationships. Interviewer bias may have influenced the findings, as the principal investigator was one of the treating dietitians. Therefore, social desirability bias may have contributed to more favourable perceptions of dietetic care, despite efforts to maintain neutrality through participant-led narratives and reflexive discussion during analysis. The small sample and post-neoadjuvant recruitment may have introduced selection bias, favouring patients with better treatment tolerance and limiting insights from those with early dropout or severe morbidity. Furthermore, COVID-19 restrictions necessitated a change to telephone interviews mid-study, and this could have affected data richness through loss of non-verbal cues. Follow-up limited to 3 months post-discharge may overlook long-term nutritional and psychosocial outcomes, as seen in extended studies [27].

## Conclusion

This study provides novel longitudinal insights into patients’ experiences of dietetics care and nutrition management during curative treatment for oesophageal and gastric cancers. Nutrition impact symptoms and weight/muscle loss were prevalent across interviews, profoundly affecting daily life, with patients relying on supplementation and support networks throughout their journey. Patients and carers demonstrated strong awareness of nutrition’s role in surgical recovery and QOL. They regarded dietitians as invaluable, offering essential guidance to navigate complications and providing psychosocial support. Participants exhibited resilience amid treatment challenges, including home enteral nutrition management. These findings endorse intensive, individualised nutrition care pathways incorporating enteral/oral supplementation to optimise nutritional status, QOL, and patient experiences in this vulnerable group. Future research should evaluate nutrition-focused peer support interventions (e.g., digital groups or randomised trials) to address psychosocial needs, with resources allocated for equitable implementation across diverse healthcare settings.

## Supporting information

S1 File COREQ Checklist. (PDF)

S2 File PG-SGA Short Form. (PDF)

S3 File Interview Schedule. (PDF)

## Abbreviations

QOL: Quality of Life
UGI: Upper Gastrointestinal
ESPEN: European Society of Clinical Nutrition and Metabolism
COREQ: Consolidated Criteria for Reporting Qualitative Research
PG-SGA Short Form: Patient-Generated Subjective Global Assessment Short Form
ICD-10-AM: International Classification of Diseases, 10th Revision Australian modification
