# Supplementary material for: “I had your number on speed dial:” A qualitative study of patient experiences of dietetics care and managing nutrition during curative treatment of upper gastrointestinal cancer
Source: PLoS One. 2026 Sep 17;21(9):e0357580. doi: 10.1371/journal.pone.0357580 (PMC13585224; doi:10.1371/journal.pone.0357580)
Supplement: Supplementary file 3 [file pone.0357580.s003.pdf]

Supplementary File 3: Interview Schedule

Interview Question Schedule Timepoint 1: Post neoadjuvant therapy.

1. Can you tell me how you think your nutrition is going currently?
   a. Prompt: Do you feel strong, weak, have you lost weight/gained weight?

2. What changes did you notice in terms of your nutrition throughout your treatment?
   a. Prompt: eating, drinking, strength, weight etc.
   b. How did you manage those changes?
   c. How did you feel during that time?

3. Can you tell me how you have been managing your nutrition at home during your treatment?
   a. Prompt: Can you tell me about any specific advice you were following during your treatment?

4. Can you tell me about your chemo/radiotherapy experience?
   a. Did the treatment impact your ability to eat or drink at all?
   b. Prompt: can you tell me about this experience?

5. Can you describe any symptoms you may have had/be having that has affected your eating or drinking?
   a. Prompt: examples include nausea, loss of appetite, diarrhea
   b. Have these symptoms been affecting your ability to do your day to day activities?
   c. What about your ability to participate in social activities (prompt if required: example includes family gatherings, going out for dinner)

6. What sorts of things have you been doing to help you with your eating and drinking?
   a. Have you changed your eating habits at all since you started treatment?

b. Has anything in particular helped you improve your eating and drinking?

7. If received a feeding tube- can you tell me about the process that lead to the insertion of the feeding tube?

8. Tell me about the experience with the feeding tube
    a. How did you feel when it was inserted?
    b. Do you feel it helped?
    c. How did you feel when it was removed?
    d. Did it affect social activities or daily tasks?
    e. How do you think you would have gone without the feeding tube?

9. If dysphagia: how did you feel when you weren't able to eat very much?
    a. Did you to anything to manage?

10. Can you tell me about your experiences seeing the dietitian(s) during your treatment?

11. What types of advice did the dietitian provide to you?
    a. Are you taking any nutritional drinks/following a specific diet?

12. How do/did you feel about this advice?
    a. Do you feel this advice has helped you during your treatment?
    b. Can you tell me if there have been any difficulties in following this advice?

13. Was there any other advice or help that the dietitian could have provided to you?

14. How important is it to you to maintain good nutrition before your surgery?
    a. Prompt: e.g. feeling strong and maintaining your weight

15. What are your thoughts on the importance of nutrition in preparation for surgery?

16. How do you feel going into surgery?

17. Do you have any other comments or questions about the nutritional care you have received?

Interview Question Schedule Timepoint 2: 3 months post surgery.

1. Can you tell me how you think your nutrition is going currently?
    a. Prompt: Do you feel strong, weak, have you lost weight/gained weight?

2. What changes did you notice in terms of your nutrition after discharge from surgery?
    a. Prompt: eating, drinking, strength, weight etc.
    b. How did you manage those changes?
    c. How did you feel during that time?

3. Can you tell me about your experiences going home after surgery?

4. Can you tell me how you have been managing your nutrition at home after surgery?
    a. Prompt: Can you tell me about any specific advice you were following after surgery?

5. Has the surgery impacted your ability to eat or drink at all? Can you tell me about this?
    a. Prompt: examples include fullness, reflux, nausea, loss of appetite, diarrhea
    b. Have these symptoms been affecting your ability to do your day-to-day activities?
    c. What about your ability to participate in social activities (prompt if required: example includes family gatherings, going out for dinner)

6. What sorts of things have you been doing to help you with your eating and drinking?

   a. Have you changed your eating habits at all since you your surgery?
   b. Has anything in particular helped you improve your eating and drinking?

7. If received a feeding tube- tell me about the experience with the feeding tube after surgery
   a. How did you feel when it was inserted?
   b. Do you feel it helped?
   c. How did you feel when it was removed?
   d. Did it affect social activities or daily tasks?
   e. How do you think you would have gone without the feeding tube?

8. Can you tell me about your experiences seeing the dietitian(s) after surgery?

9. What types of advice did the dietitian provide to you?
   a. Are you taking any nutritional drinks/following a specific diet?

10. How do/did you feel about this advice?
    a. Do you feel this advice has helped you after surgery?
    b. Can you tell me if there have been any difficulties in following this advice?

11. Was there any other advice or help that the dietitian could have provided to you?

12. How important is it to you to maintain good nutrition after your surgery?
    a. Prompt: e.g. feeling strong and maintaining weight

13. Do you have any other comments or questions about the nutritional care you have received?
